# Microbial community diversity and potential functionality in response to dam construction along the Three Gorge Reservoir, China

**DOI:** 10.3389/fmicb.2023.1218806

**Published:** 2023-09-20

**Authors:** Huan Wang, Bin Yan, Yan Wu, Maoyun Yin, Maoqing Wang, Chuan Fu

**Affiliations:** ^1^Chongqing Key Laboratory of Water Environment Evolution and Pollution Control in Three Gorges Reservoir, Chongqing Three Gorges University, Wanzhou, China; ^2^Chongqing Landscape and Gardening Research Institute, Chongqing, China; ^3^Chongqing Key Laboratory of Germplasm Innovation and Utilization of Native Plants, Chongqing, China

**Keywords:** bacterial community, biodiversity, functionality, microbial ecology, Three Gorges Reservoir

## Abstract

River and reservoir bacterial communities are the most basic part of river biomes and ecosystem structure, and play an important role in river biological processes. Yet, it remains unclear how highly regulated dam reservoirs affect both soil and sediment bacterial communities. A temporal distribution pattern of bacterial communities was investigated using Illumina MiSeq sequencing in a transition section of the Three Gorges Reservoir (TGR). In total, 106,682 features belong to the bacteria kingdom, encompassing 95 phyla, 228 classes, 514 orders, 871 families, 1959 genera, and 3,053 species. With water level regulation, Shannon diversity index, and observed species differed significantly, with no significant difference in Simpson evenness. Both in the high water level period (October) and the low water level period (June), Proteobacteria, Acidobacteri, and Chloroflexi were the most abundant phyla. Whereas, based on PCA plots and Circos plot, the microbial community structure has changed significantly. LEfSe method was used to identify the classified bacterial taxa with significant abundance differences between the low water level and high water level periods. KOs (KEGG Orthology) pathway enrichment analysis were conducted to investigate functional and related metabolic pathways in groups. To some extent, it can be inferred that water level regulation affects community growth by affecting the metabolism of the microbial community.

## 1. Introduction

A healthy ecosystem depends on the function and health of microorganisms (Niu et al., 2019; Wagg et al., 2019). Firstly, microorganisms are considered to be the main drivers of the global biogeochemical cycle and the main decomposers in terrestrial and aquatic ecosystems. Soil microorganisms play an irreplaceable regulatory role in the process of material and energy cycling in soil ecosystems, and they are often used as important parameters to assess soil activity (Zhou and Wang, 2015). Therefore, a full understanding of the changing characteristics and roles of microbial communities in soil ecosystems is of great practical significance to the evolution of soil quality, the soil carbon cycle, the sustainable development of agriculture, global climate change, as well as the restoration and reconstruction of degraded ecosystems (Gao et al., 2017; Chunhua et al., 2022).

Through the confluence of surface runoff and tributaries, rivers interact closely with a variety of ecosystems (Ahn and Grant, 2007). Microorganisms from different habitats also continue to flow into the river, creating the characteristics of high microbial diversity in the river ecosystem (Staley et al., 2016). However, the research of river microorganisms is much rarer than that of terrestrial, lake and marine ecosystems (Xu et al., 2012; Guerra et al., 2020; Qin et al., 2021). The world’s oceans comprise a rich diversity of microbial life with current estimates reaching over a million different species (Whitman et al., 1998). River and reservoir bacterial communities are the most basic part of river biomes and ecosystem structure, with rich variety, diverse functions and responsiveness, and play an important role circulation and nutrient conversion in river, organic matter formation and decomposition, global material circulation and energy flow, and other biological processes (Sommers et al., 2019; Qin et al., 2021). Microorganism are the main driver of river material transformation and energy flow, and it is the key factor to improve the self-purification ability of river water environment and the self-healing ability of water ecology. The construction and succession process of river microbiome reflects the relationship between microorganism and river ecosystem process, function, resilience and sustainability, and is the key link of the law of the migration and transformation of pollutants in rivers. Therefore, the use of microbial information to assess the current situation of the river ecological environment, analysis the causes of degradation, trace the source of pollution is an important prerequisite for the comprehensive management of watershed water environment, the reconstruction of river ecosystem is of great significance. Fluctuating water level changes in the riparian zone lead to subsequent changes in soil moisture content. Moisture is the most important influence in riparian zone ecosystems (Regnery et al., 2020). Microbial community structure and function and soil moisture show a consistent trend within a certain range. Under the fluctuating water level, the type of vegetation covered also changed, which led to different quality and quantity of apoplastic materials, and also caused changes in soil physicochemical properties, which directly or indirectly affected soil microbial composition and function (Weihua et al., 2022). Therefore, the use of microbiological information is an important means of assessing the status of river ecosystems, analysing the causes of degradation and tracing the sources of pollution. This is of great significance for river ecosystem management and protection.

Located between Chongqing and Three Gorges Dam (TGD), the Three Gorges Reservoir (TGR) is the world’s largest deep river-type reservoir, covering an area of 1,084 km^2^, and it extends over 670 km (Chen et al., 2013). Periodical fluctuations in the water level directly impact the riparian zone of the TGR, which is impounded at 175 meters in October and discharged at 145 meters in March for flood control in one consecutive inundation cycle (Wang et al., 2019). Wanzhou district is the largest city in TGR with a population of 800,000 people (Xiao et al., 2013). Wanzhou district is about 288 km away from the Three Gorges Dam, and the highest water level is relatively stable throughout the year, basically remaining at 175 m, while the lowest water level basically remains at 145 m. Due to the special environment of the TGR area, research on the microbial diversity of the TGR area have attracted attention, including environmental factors and microbial communities in backwater and riverine habitats (Niu et al., 2019; Qin et al., 2021). Most studies have focused on the hydrological environment and reservoir bank plant communities in transition zones. Most of publications focused on the communities. Typical transition zones, however, remain largely untouched by the reactions of soil and sediment bacterial communities to environmental disturbances. Species diversity, functions, and associations of bacterial consortia are still unknown, therefore more intensive, comprehensive, and fundamental investigations are needed.

The aim of this study is to gain more insight into the nature and form of association and interaction in bacterial assemblages, using high-throughput amplicon sequencing of genes associated with 16S rDNA, and we characterized the bacterial assemblages associated with 90 samples that have been collected during different periods (high and low water levels in TGR). Therefore, the main objectives of this study were to diversity of bacterial communities potentially associated with water levels and to characterize the bacterial communities. Based on the results obtained, a meaningful understanding of the species composition and community functional profiles of bacterial assemblages in TGR is expected to be gained.

## 2. Materials and methods

### 2.1. Study sites, grouping details, and sampling

**Figure 1:**
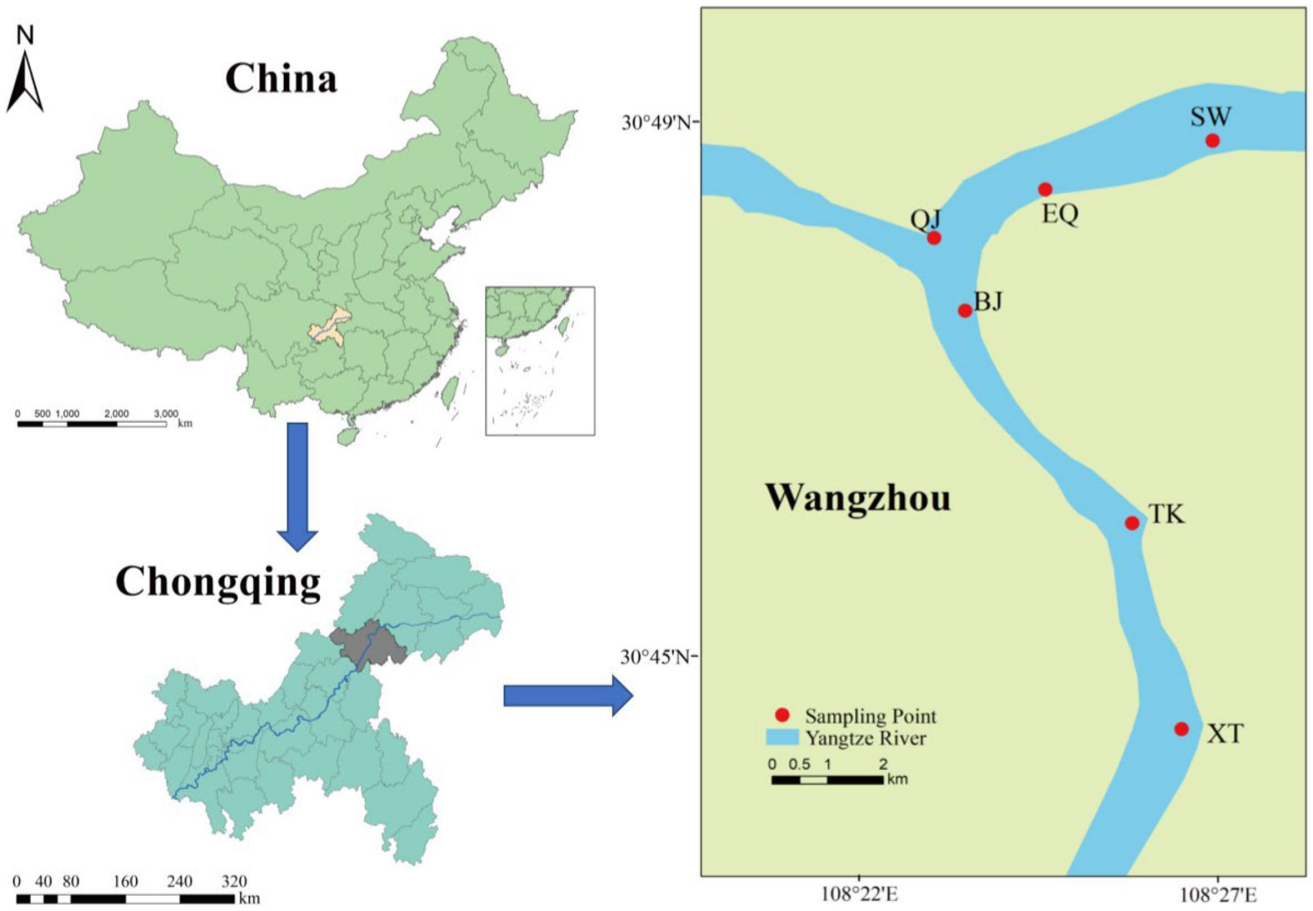
Map of the locations of the sampling sites. The sampling locations were XT, TK, BJ, EQ, QJ, and SW.

The sampling sites of this research generally cover the largest deep-water reservoir in the Three Gorges reservoir across Wanzhou (Figure 1). Soil and sediment samplings were synchronously conducted in June (low water level at 165 m), and October (high water level at 175 m) of 2020, and the sample site positions are same in June and October. A total of 90 samples were collected, including 54 soil samples and 36 sediment samples. There are a total of six stations, including Xintian (XT), Tuokou (TK), Bingjiang (BJ), ChangjiangErqiao (EQ), Qingjiang (QJ), and Shaiwangba (SW). The samples with low-water level samples in June were grouped D and with high-water level samples in October grouped G. In group D, samples from the same site included samples at different altitudes, including sediment samples below 165 m grouped ND, soil samples between 165–175 m grouped TD1, and soil samples above than 175 m grouped TD2. Group G includes sediment samples at altitudes between 165–175 m (grouped NG) and soil samples above 175 m (grouped TG). Three samples were collected from each sampling site in different periods. Sample information was provided in Supplementary Table S1. Sediment samples were collected using a dredger and box-type mud extractor, and stored in sterilized polypropylene tubes in coolers for microbial analysis, and then transported to the laboratory.

### 2.2. DNA extraction, PCR amplification, and Illumina MiSeq sequencing

As directed by the manufacturer, genomic DNA was extracted from each sample using the E.Z.N.A.^®^Soil DNA Kit. Total DNA was eluted with 50 μL TE buffer (Tris-hydrochloride buffer, pH 8.0, 1.0 mM EDTA contained). In order to determine DNA concentration and purity, a NanoDropTM 1000 spectrophotometer (Thermo Fisher Scientific, Waltham, MA, United States) was used and samples were stored at −80°C until PCR amplification. PCR amplification of the V3–V4 region of the 16S ribosomal RNA gene was carried out on bacteria (98°C for 30 s; with 35 cycles at 98°C for 10 s, 54°C for 30 s, and 72°C for 45 s; and a final extension at 72°C for 10 min) using a primer set of 341F (5’-CCTACGGGNGGCWGCAG-3′) and 805R (5’-GACTAC HVGGGTATCTAATCC-3′) (Logue et al., 2016). Barcodes were added to the 5′ ends of the primers and universal primers were used. Each PCR reaction was conducted in a 25 μL mixture containing 12.5 μL of 2× Phusion^®^ Hot Start Flex Master Mix, 2.5 μL of each primer (1 μM), and 50 ng of template DNA. Nuclease-free water served as blank. For the DNA extraction process, ultrapure water was used instead of a sample solution to ensure that there were no false positives. Throughout the DNA extraction process, ultrapure water was used in place of template DNA as a negative control to exclude false-positive PCR results. 2% agarose gel electrophoresis was used to verify the PCR amplicon size. PCR products were purified using AMPure XT beads from Beckman Coulter Genomics in Danvers, MA, United States and quantified using Qubit from Invitrogen, United States. The amplicon library was sized and quantified using an Agilent 2100 Bioanalyzer (Agilent, United States) and a Library Quantification Kit for Illumina (Kapa Biosciences, Woburn, MA, United States), respectively. A NovaSeq PE250 platform at LC-Bio Technology Company (Hangzhou, China) was used to sequence the libraries.

### 2.3. Sequencing data processing and bioinformatic analyses

Based on samples’ unique barcodes, paired-end reads were assigned, then truncated by cutting off the barcodes and primer sequences, and merged with FLASH (Tanja and Salzberg, 2011). Fqtrim software was used to trim and filter the raw reads, and Vsearch software was used to further filter the chimeric reads (Torbjørn et al., 2016). The amplicon sequence variants (ASVs) were generated with DADA2 package (Callahan et al., 2016). Through BLAST searches, all of the sequence reads were compared against the Silva rRNA database (Christian et al., 2013). Using the average abundance of each group, the relative abundance of each taxon was calculated by normalizing assigned reads to the total number of qualified reads. The rarefaction curves were generated using custom Perl scripts for each sample. The BioVenn software was used to plot Venn diagrams showing the shared and unique features (http://www.biovenn.nl/index.php accessed on 2 November 2022). In order to analyze the complexity of species, the alpha diversity indices (Observed species, Shannon diversity, and Simpson evenness) were calculated using QIIME 2 (Quantitative insights into microbial ecology 2) (Bolyen et al., 2019). One-way ANOVA or *t*-tests was used to test the significance of variance between or among samples using SPSS 22.0 (SAS Institute Inc., Cary, NC, United States). An analysis of beta diversity was used to display and compare bacterial community compositions. With the QIIME 2 plugin, PCA (principal component analysis) was conducted (Bolyen et al., 2019). Using the group average method, hierarchical agglomerative clustering (to group objects similar to each other in clusters) was carried out on the most abundant features according to the groups selected. With the help of OmicStudio tools, we measured the LDA (Linear discriminant analysis) effect size and coupled it with standard statistical tests to determine the observed characteristics most likely to explain differences between samples (Nicola et al., 2011). By using the OmicStudio tool, a heatmap of bacterial communities was generated, with Bray–Curtis similarity calculations used to cluster relative abundance data (Polson, 2007). Unless otherwise stated, all statistical analyses were conducted at a significance level of 0.05.

### 2.4. Functional annotation of the presented common bacterial communities

PICRUSt2 (Phylogenetic Investigation of Communities by Reconstruction of Unobserved States) algorithm was used to predict the metagenomes of the bacterial communities of different groups, in order to explore potential functional differences between the communities (Douglas et al., 2020). The PICRUSt algorithm was used to make inferences from the KEGG database about bacterial gene functions using 16S rRNA gene-based species compositions for bacteria (Langille et al., 2013). Gene families and pathways that showed differential abundance were identified using STAMP software and analyzed with *t*-tests and Tukey–Kramer *post hoc* analyses with Benjamini–Hochberg FDR multiple comparison correction (Parks et al., 2014). When *p* < 0.05, a significant difference was inferred.

## 3. Results

### 3.1. Sequence data results and community composition

**Figure 2:**
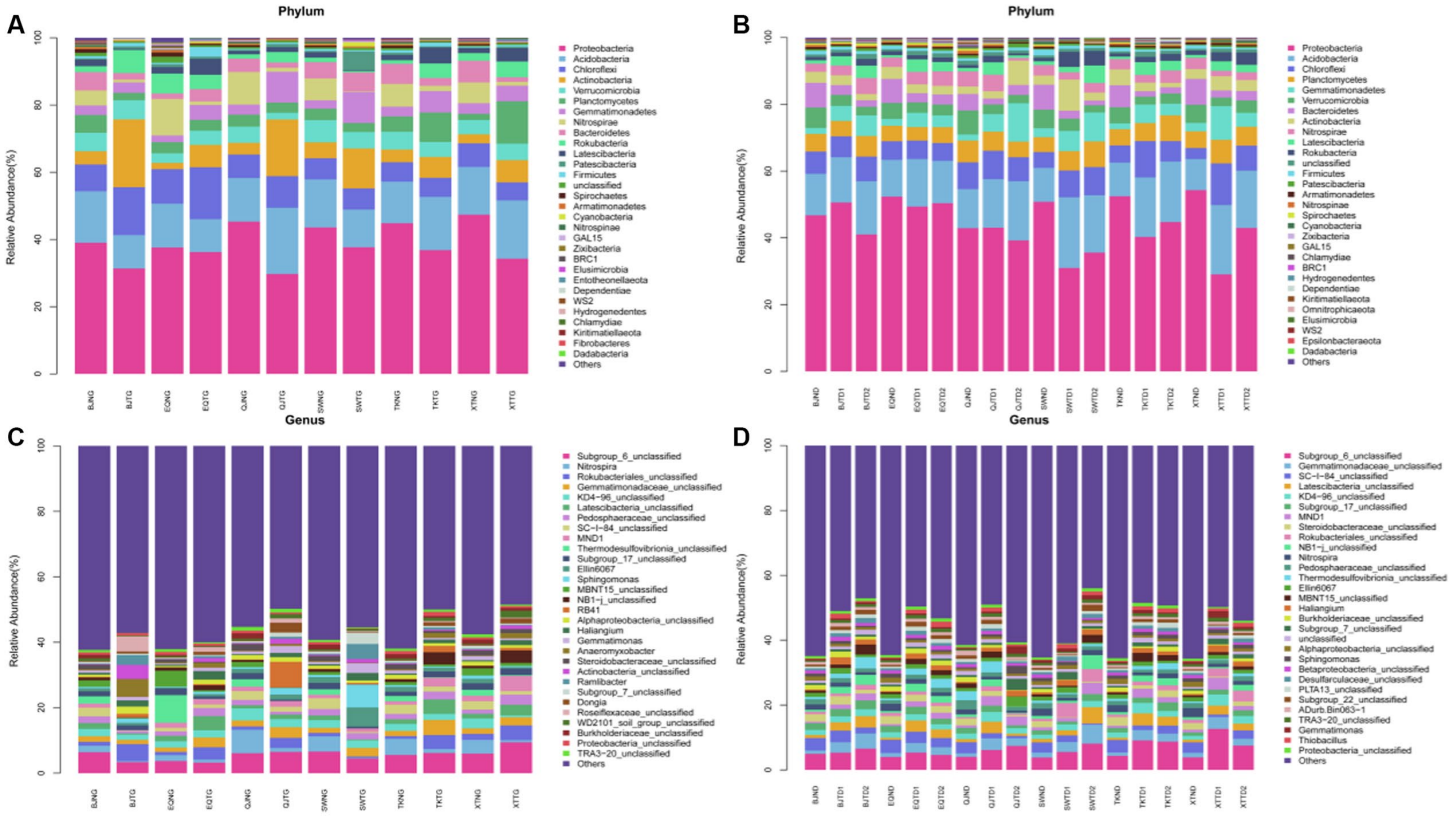
Relative abundance of different phyla **(A**,**B)** and genera [top 30; **(C**,**D)**] in the samples, **(A**,**C)** were in period of high water level, and **(B**,**D)** were in period of low water level. The abundance is presented in terms of percentage in total effective features in a sample.

With an average of 84,140 sequences per sample, the 90 samples resulted in 7,572,584 raw rDNA sequence reads, which equal 3.70 Gb of raw data. The final sequences were approximately 2.61 Gb in size, with 84.26% quality control efficiency after removing poor quality, short, and chimeric sequences (Supplementary Table S2). After dereplication with DADA2 within QIIME2, 106,682 prokaryotic features were obtained. As sequencing amount increased, all rarefaction curves reached saturation, with a mean Good’s coverage of 0.84 (Supplementary Table S3) for all samples. A sufficient sequencing depth is required to reveal the species diversity of samples associated with prokaryotes. By blasting against the database Silva 128, all 106,682 effective features were categorized into different taxonomical levels from kingdom to species (https://www.arb-silva.de/accessed on 13 July 2020 and 20 October 2020). The most features belonging to the bacteria (99.14%). The features were assigned to 95 phyla, 228 classes, 514 orders, 871 families, 1959 genera, and 3,053 species (Supplementary Table S4). Among 90 samples, there were 187 to 5,127 features (mean = 2,687) (Supplementary Table S4). Proteobacteria (41.63%) were the most abundant phylum of bacteria, followed by Acidobacteria (13.81%), Chloroflexi (7.84%), and Planctomycetes (4.98%). At the genus level, Subgroup_6_unclassified (5.91%), *Gemmatimonadaceae*_unclassified (2.98%), SC-I-84_unclassified (2.15%), KD4-96_unclassified (2.13%), and *Latescibacteria*_unclassified (2.02%) were the top 5 abundant genera, and these features accounted for 15.19% of the entire collection, while “unclassified” represents sequences marked as “unclassified bacteria.” The most three abundant microbial phylum were Proteobacteria, Acidobacteria, and Chloroflexi both in high and low water level periods (Figures 2A,B). The most abundant microbial genus were Subgroup_6_unclassified (5.63%), Nitrospira (2.44%) and Rokubacteriales_unclassified (2.34%) in high water level periods showed in Figure 2C. The most abundant microbial genus were Subgroup_6_unclassified (6.23%), Gemmatimonadaceae_unclassified (2.60%), and SC-I-84_unclassified (2.51%) in low water level periods showed in Figure 2D.

### 3.2. Species composition of bacterial communities during different period

**Figure 3:**
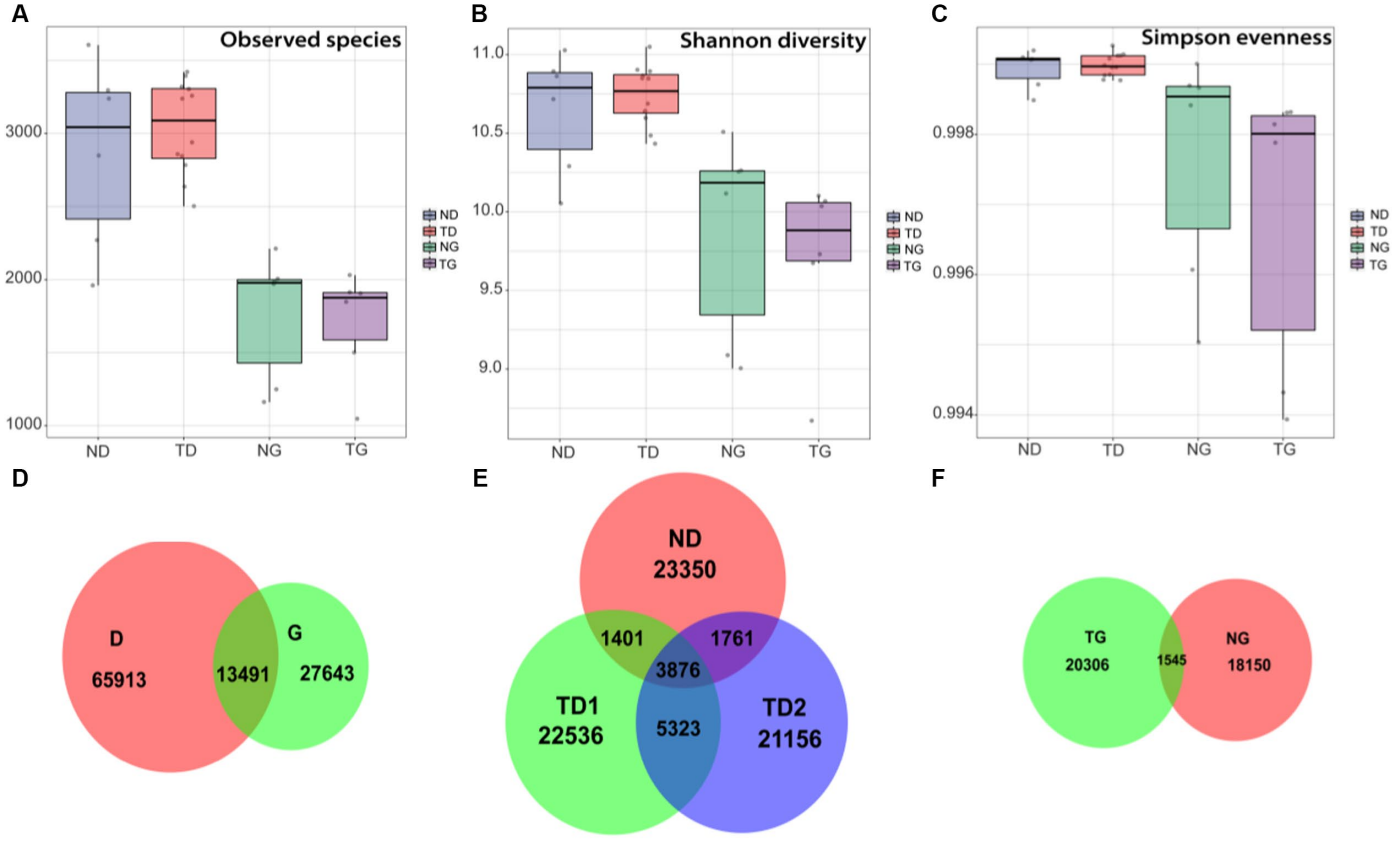
Box plots (median, min and max) showing alpha diversity [the number of observed species **(A)**, Shannon diversity **(B)** and Simpson evenness **(C)**] of ND (sediment-low water level), TD (soil-low water level), NG (sediment-high water level) and TG (soil-high water level) groups. Venn diagram showing the numbers of shared and unique bacterial features. **(D)**: low and high water level groups, **(E)**: soil and sediment samples of low water level groups, **(F)**: soil and sediment samples of high water level groups.

Comparing the high level water period (36 samples) and low level water period (54 samples) groups, It was found that Shannon diversity and Observed species differed significantly (ANOVA, *p* < 0.05; Figures 3A,B), with no significant difference in Simpson evenness (ANOVA, *p* > 0.05; Figure 3C). Venn diagram showed that D and G groups share 13,491 bacterial characteristics, while have 65,913 and 27,643 unique features, respectively (Figure 3D). In low water level period, ND, TD1, and TD2 groups shared 3,876 bacterial features in common, and contained 23,350,22,536 and 21,156 unique features, respectively (Figure 3E). In high water level period, NG and TG groups shared 1,545 bacterial features in common, and contained 20,306 and 18,150 unique features, respectively (Figure 3F).

**Figure 4:**
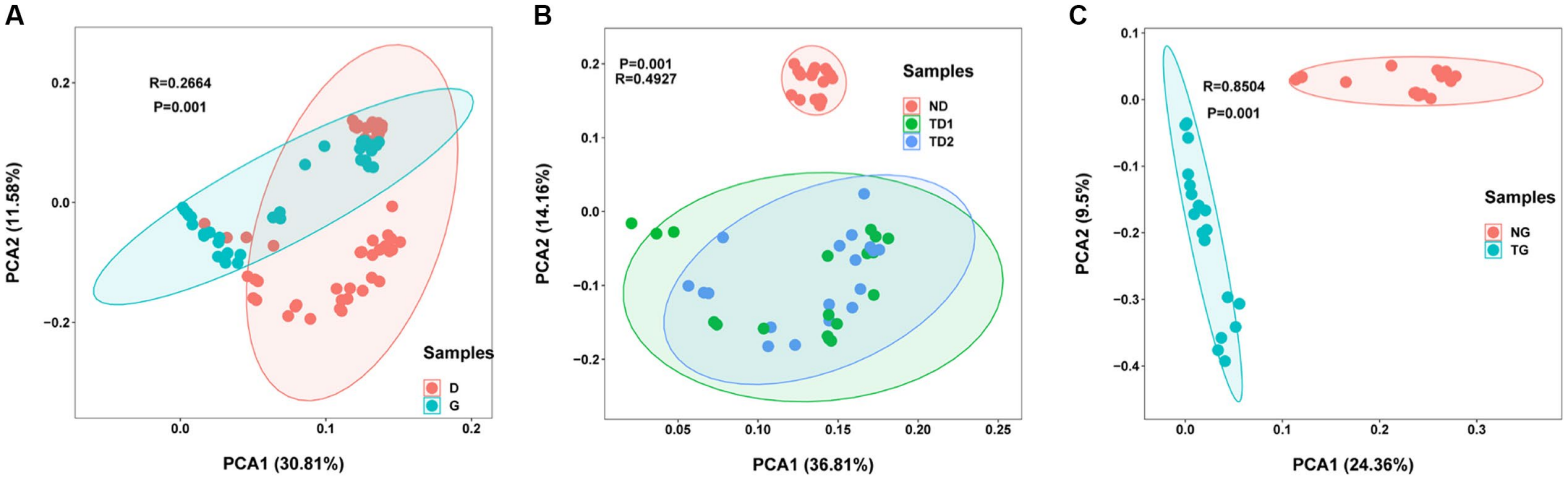
Principal component analysis (PCA) using genus/species-level Hellinger transformed relative abundances of bacterial sequences. **(A)**: low and high water level groups, **(B)**: soil and sediment samples of low water level groups, and **(C)**: soil and sediment samples of high water level groups.

In addition, beta diversity analyses were conducted to visualize similarity and dissimilarity in species (features; 100% identity) complexity among different groups. According to the PCA plots based on the weighted-uniFrac distance, group D differs from group G (*p* < 0.05, Figure 4A), the first and second axes accounted for 42.39% of the variance. For different period groups, there were significant differences between ND, TD1, and TD2 (*p* < 0.05, Figure 4B), as similar to NG and TG (*p* < 0.05, Figure 4C).

**Figure 5:**
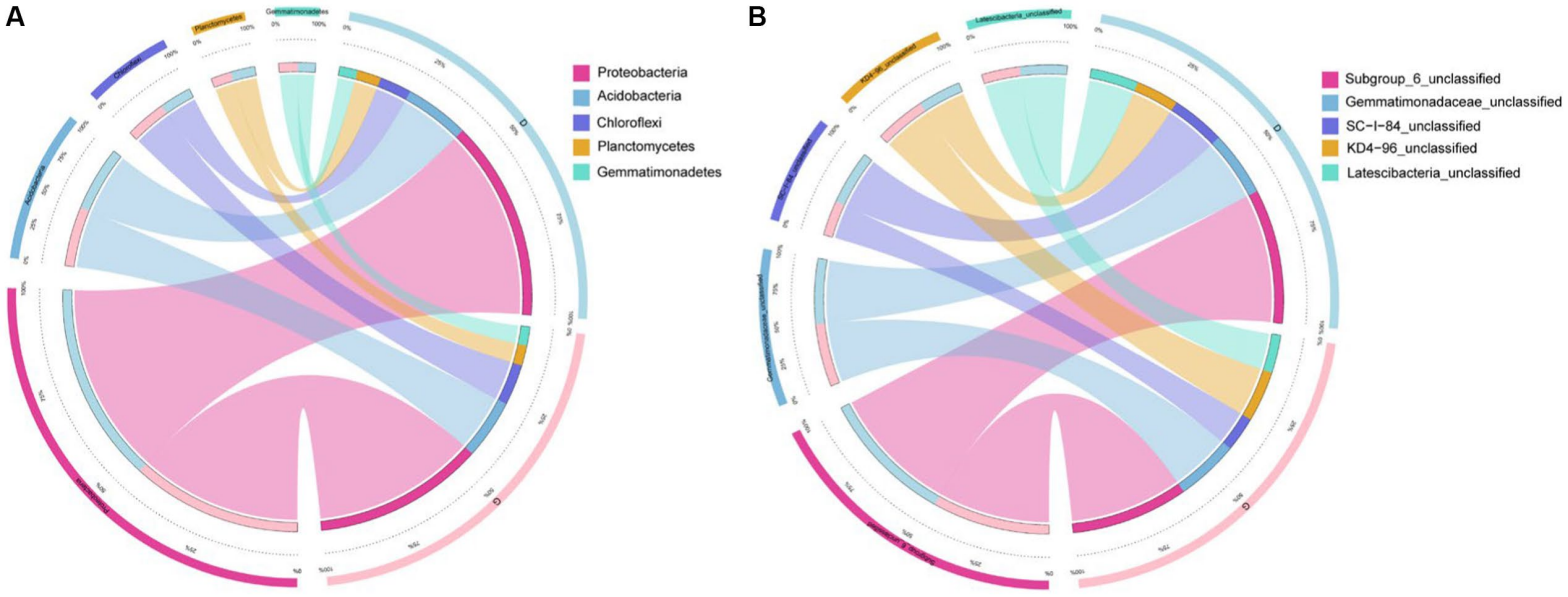
Circos plot of the top 5 abundant bacterial features at the phylum **(A)** and genus **(B)** levels in low water level groups and high water level groups.

The Circos plot reflected the composition proportion and the distribution proportion of each dominant species among D and G groups in phylum (Figure 5A) and genus (Figure 5B), the top 5 predominant phylum (Figure 5A) and genus (Figure 5B). In groups D and G, Chloroflexi was the most differentiated phylum between the two groups, and SC-I-84 unclassified was the most differentiated genus between the two groups.

### 3.3. Communities of microbes differ significantly

**Figure 6:**
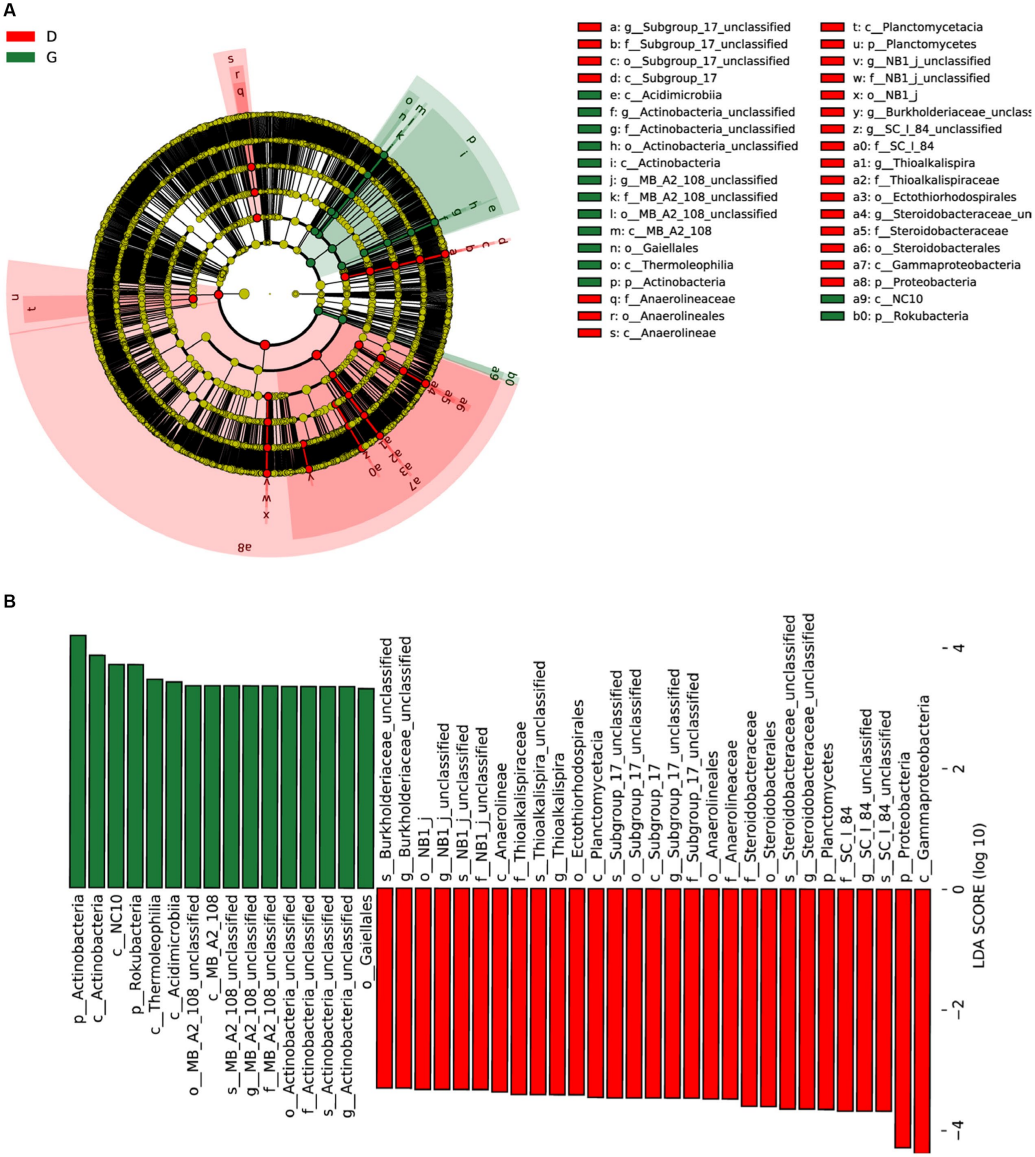
Linear discriminant analysis (LDA) integrated with effect size (LEfSe). **(A)** Cladogram illustrating the phylogenetic distribution of bacterial taxa differentially represented between low water level period (red) and high water level period (green) groups. **(B)** The differences in abundance of represented taxa between low water level period (red) and high water level period (green) groups.

Biomarker analysis was used to identify the classified bacterial taxa with significant abundance differences between the D and G groups by using the linear discriminant analysis (LDA) effect size (LEfSe) method. Most bacteria were significantly enriched in D group, while only 4 clades showed abundance in G group samples. At class level, LEfSe analysis indicated that Subgroup_17, Anaerolineae, Planctomycetacia, and Gammaproteobacteria were discriminating taxonomic units in D group. However, Acidimicrobiia, Actinobacteria, MB_A2_108, Thermoleophilia and NC10 were discriminating taxonomic units in G group at class level (Figure 6A). With a LDA threshold of 3.5, 45 bacterial clades present statistically significant differences (Figure 6B).

### 3.4. Functional genera and enzymes in microbial communities

**Figure 7:**
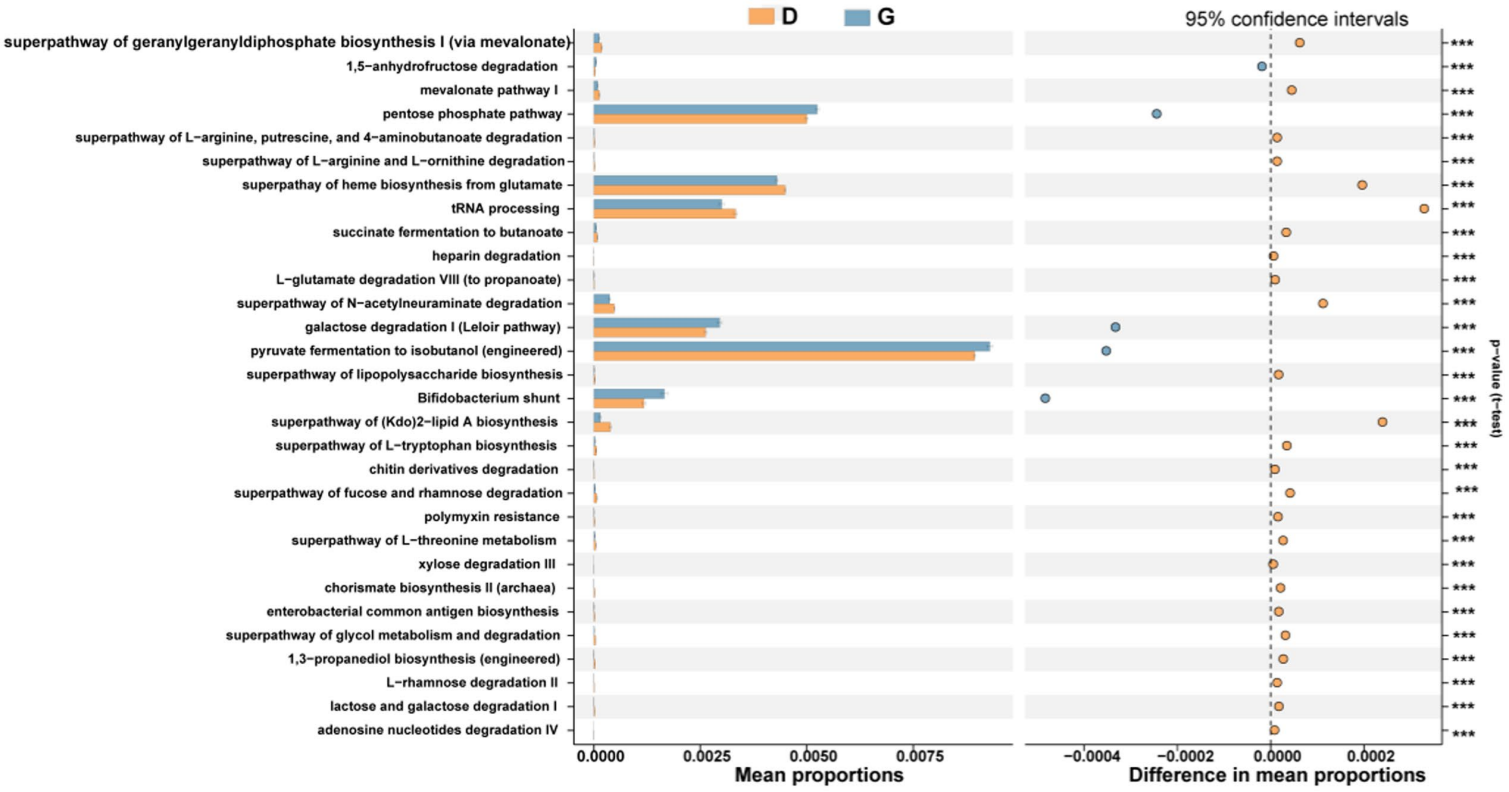
Prediction of the differential function of bacterial communities between high (blue haze) and low water level period (orange) groups in KEGG categories. Gene functions were predicted from 16S rRNA gene-based microbial compositions using the PICRUSt2 algorithm to make inferences from KEGG annotated databases. Relative signal intensity was normalized by the number of the genes for each indicated metabolic pathway. *, **, and *** indicate the difference is at a significant level with *p* < 0.05, *p* < 0.01, and *p* < 0.001, respectively.

Functional predictions were performed using PICRUSt2 to explore the ecological roles played by bacteria. For the functional modules (KEGG Orthology), 30 categories of functions were predicted. There were the top 5 enriched proportions, including Pyruvate fermentation to isobutanol (engineered), pentose phosphate pathway, superpathway of heme biosynthesis from glutamate, tRNA processing and galactose degradation I (Leloir pathway) in whole samples. In the G group, there are five categories of functions (replication and repair, transcription, genetic information processing, poorly characterized, glycogen biosynthesis, glycan metabolism, biosynthesis of other secondary metabolites, cellular processes and signaling, transport and catabolism, signal transduction and cell motility) markedly enriched. D group had significantly more relative abundances of other 25 functions (Figure 7).

## 4. Discussion

Microbial communities are fundamental in the functioning of river ecosystems. Through the confluence of surface runoff and tributaries, rivers have close interactions with various ecosystems. Microbial groups from different habitats also continue to flow into rivers, creating a high diversity of microorganisms in river ecosystems (Ahn and Grant, 2007). However, it is an important and unresolved question in microbial ecology what determines the diversity and function of microbial ecosystems (Sergei and Kim, 2017). The purpose of this study is to provide critical comparative information about bacterial communities in sediment and soil samples collected from the riparian zone of the TGR, confirming difference in diversity and temporal patterns between the sediment and soil habitats. An increasing number of studies based on high-throughput sequencing technology have demonstrated that there is communities harbored in aquatic environments (Sergei and Kim, 2017; Sommers et al., 2019). Some research has shown that complex and dynamic influence of seasonal water level regulation on microbial community in aquatic systems (Ahn and Grant, 2007). Based on high-throughput 16S rRNA gene amplicon sequencing, 90 samples from soil and sediment were analyzed for diversity and composition of bacterial associations. Generally, Proteobacteria categorically predominated (41.63%) the associate community, with Acidobacteri as sub-dominant (13.81%), followed by Chloroflexi (7.84%), and then Planctomycetes (4.98%) being relatively common at phylum level. Our results are consistent with most previous studies. Proteobacteria, as the indicators of freshwater bacteria, is widely detected in freshwater (Newton et al., 2011). It has been reported that Proteobacteria and Actinobacteria can contribute to more than 50% of the total bacteria in surface water (Yu et al., 2014).

However, the most abundant microbial genus in periods with high water level were Subgroup_6_unclassified (5.63%), *Nitrospira* (2.44%), and *Rokubacteriales*_unclassified (2.34%), and the most abundant microbial genus in periods with low water level were Subgroup_6_unclassified (6.23%), *Gemmatimonadaceae*_unclassified (2.60%), and SC-I-84_unclassified. Meanwhile, previous works on riparian bacteria communities found that the difference in microgeochemical environments and the influence of the regulated dam may explain the diversity of bacterial communities in the water and sediment (Cole et al., 2013; Su et al., 2022). The most common bacterial phyla was Acidobacteria both in soils and sediments, followed by Proteobacteria, instead of Proteobacteria and Actinobacteria, with over 50% of the total. In previous studies, acidic environments and polluted sites were found to have a higher prevalence of Acidobacteria (Fabienne et al., 2003). The specific phylum Acidobacteria dominated in the microbial community along the contaminated watershed in previous study (Wang et al., 2018). It is inferred that the water bodies in the Three Gorges reservoir area has been polluted to some extent. As a result of a genomic study, Acidobacteria can use a wide variety of carbohydrates, as well as both organic and inorganic nitrogen (Eichorst et al., 2018). Organic matter decomposition and nutrient cycling were also carried out by actinobacteria (Gil-Martinez et al., 2020). These samples contained 106,682 features belonging to the bacteria kingdom, including 95 phyla, 228 classes, 514 orders, 871 families, 1959 genera, and 3,053 species. In this study, the taxonomic statistical data is larger than that of general fluctuation zone survey (Niu et al., 2019; Qin et al., 2021).

An analysis of the alpha diversity, beta diversity, and temporal distribution patterns of bacterial communities was conducted on soils and sediments. Significant differences were detected in Shannon diversity and Observed species in different samples periods (ANOVA, *p* < 0.05; Figures 3A,B). Although environmental parameters are not widely measured in this study, our belief remains that a close relationship exists between bacterial community distribution and physiochemical characteristics (Pei et al., 2018; Niu et al., 2019). The average number of soil features was 42,153 (21,847 for low water level and 20,306 for high water level), and the average number of sediment features was 42,500 (23,350 for low water level and 18,150 for high water level). Some research suggests that soil microbiomes are more diverse in soils flooded with water than before stress occurred (Furtak et al., 2020; Jinyan et al., 2022). Nevertheless, some studies have confirmed rapid flooding, with a significant reduction in soil bacterial a diversity after flooding (Gvozdikova et al., 2019; Furtak et al., 2020). The speed of inundation may be a non-negligible factor affecting the diversity of soil bacteria. The Three Gorges Dam impoundment was a short-term human behavior, and the inundation rate was rapid, resulting in a decrease in the number of soil bacterial organisms after inundation. The soil bacterial community was not more sensitive to variations in water level than the sediment bacterial community. Although there is no significant change in feature number, it can be inferred from the change of biodiversity indices that the microbial community structure has changed significantly. PCA plots and Circos plot also reflected clearly the composition proportion and the distribution changes among different water level periods. The biodiversity of microbes in a river can change significantly due to intense environmental changes resulting from intense environmental changes in different environments (Gvozdikova et al., 2019). Therefore, researchers have increasingly focused on understanding how communities are assembled in riverine environments (Jiao et al., 2020). A comprehensive understanding of microbial community diversity, functions, and biogeography is crucial to understanding ecological succession (Zhou et al., 2014). The between-group differences in PCA analysis between sediment and soil (*R* values equal to 0.4927 and 0.9504, respectively) was significantly higher than the total between-group differences between high and low water level (*R* values equal to 0.2664), and these results were consistent with other findings that frequent flood disturbances lead to an alternate microbial structure (Gvozdikova et al., 2019).

LEfSe analysis supported Thioalkalispira, *Steroidobacteraceae*-unclassfied, and *Burkholderiaceae*-unclassfied being three of the discriminating feature between D and G groups. In 2002, *Thioalkalispira*, a novel gamma-Proteobacterium genus from a soda lake, was described for the first time. It represents a new genus within the *gamma-Proteobacterium* family (Sorokin et al., 2002). *Thioalkalispira microaerophila* is proposed as the type species of this novel genus. *Thioalkalispira* and *steroidobacteraceae* were members belonging to γ-proteobacteria, and *Burkholderiaceae* was member belonging to β-proteobacteria. As the water level fluctuates, these biomarker bacteria played an important role in the cycle of nitrogen elements such as the degradation of dissolved organic matter, the reduction of NO_2_^+^ to NO, the degradation of organic nitrogen, the reduction of NO_3_^+^ to NO_2_^+^, and the utilization of dissolved organic nitrogen, suggesting that these bacteria in the sediment and soil of the Three Gorges Reservoir can affect the biological cycle of nitrogen in water (Watanabe et al., 2009; Orsi et al., 2016).

Microbial function processes are compound and closely related to environmental conditions and microbial physiological status. The PICRUSt function prediction analysis based on high-throughput sequencing has been world-widely used in the research of oceans, lakes, soil microorganisms and other habitats due to its low cost, convenience and reliability (Deng et al., 2022; Yin et al., 2023). Additionally, we observed an enrichment of KOs (KEGG Orthology) pertaining to nutritional metabolic function in groups. For the functional modules (KEGG Orthology), Pyruvate fermentation to isobutanol (engineered), pentose phosphate pathway, superpathway of heme biosynthesis from glutamate, tRNA processing and galactose degradation I (Leloir pathway) were the top 5 enriched proportions. Pyruvate fermentation to isobutanol, pentose phosphate pathway, superpathway of heme biosynthesis from glutamate and galactose degradation I were involved in the aerobic oxidation of glucose for energy supply. It can be inferred that water level fluctuation has a certain degree of stress effect on the three major nutrients (protein, nucleic acid, sugar) of soil and sediment microorganisms, which is reflected in the expression of functional genes. In terms of genetic information processing, aminoacyl-tRNA biosynthesis and ribosomes are related to translation, while RNA polymerase is related to transcription (He et al., 2015).

## 5. Conclusion

To understand the bacterial communities in large reservoir ecosystems, it is essential to understand soil and sediment bacterial communities. Using soil and sediment samples from the TGR as a comparative sample, our study provides valuable information about bacterial communities, evidencing a distinction between the two habitats in terms of biodiversity and temporal patterns. The results of diversity index and grouping difference analysis showed that the microbial community structure changed significantly in different periods. Fluctuations in water levels affect the three major nutrients (protein, nucleic acid, sugar) of soil and sediment microorganisms, which are reflected in gene expression. All in all, our findings provide some vital information and evidence towards a better understanding of microbial abundance and diversity as well as fundamental functions of bacteria in river ecosystems of the TGR.

## Data availability statement

The data presented in the study are deposited in the NCBI repository, accession number PRJNA929072.

## Author contributions

HW, BY, and CF: conceptualization, methodology. HW and YW: formal analysis. HW and CF: funding acquisition. HW and MW: investigation. HW: writing – original draft. YW, MW, and MY: writing – review and editing. All authors contributed to the article and approved the submitted version.

## Funding

This work was supported by Postdoctoral Science Foundation of Chongqing Natural Science Foundation (Grant No. CSTB2022NSCQ-BHX0740); the National Natural Science Foundation of China (Grant No. 31670467); the Performance Incentive and Guidance Special Project for Chongqing Scientifific Research Institutes (No. cstc2022jxjl20010); the Scientifific research project of Chongqing City Administration Bureau (No. CGK 2022-19); the Science and Technology Research Program of Chongqing Municipal Construction Commission (NO. CKZ2019-2-3-2); The Science and Technology Innovation Project of Chongqing (Grant No. cstc2021jscx-xczx0014).

## Conflict of interest

The authors declare that the research was conducted in the absence of any commercial or financial relationships that could be construed as a potential conflict of interest.

## Publisher’s note

All claims expressed in this article are solely those of the authors and do not necessarily represent those of their affiliated organizations, or those of the publisher, the editors and the reviewers. Any product that may be evaluated in this article, or claim that may be made by its manufacturer, is not guaranteed or endorsed by the publisher.
